# The anthelmintic praziquantel is a human serotoninergic G-protein-coupled receptor ligand

**DOI:** 10.1038/s41467-017-02084-0

**Published:** 2017-12-05

**Authors:** John D. Chan, Pauline M. Cupit, Gihan S. Gunaratne, John D. McCorvy, Yang Yang, Kristen Stoltz, Thomas R. Webb, Peter I. Dosa, Bryan L. Roth, Ruben Abagyan, Charles Cunningham, Jonathan S. Marchant

**Affiliations:** 1 Department of Pharmacology, University of Minnesota, Minneapolis, MN 55455 USA; 2 Skaggs School of Pharmacy and Pharmaceutical Sciences, University of California San Diego, La Jolla, CA 92093 USA; 3 Department of Pharmacology, University of North Carolina at Chapel Hill, Chapel Hill, NC 27599-7365 USA; 4 Institute for Therapeutics Discovery and Development, University of Minnesota, Minneapolis, MN 55414 USA; 5 Division of Biosciences, SRI International, Menlo Park, CA 94025 USA; 6 Division of Chemical Biology and Medicinal Chemistry, Eshelmann School of Pharmacy, University of North Carolina at Chapel Hill, Chapel Hill, NC 27599-7360 USA; 7 National Institute of Mental Health Psychoactive Drug Screening Program (NIMH PDSP), School of Medicine, University of North Carolina at Chapel Hill, Chapel Hill, NC 27599-7360 USA; 8 Department of Biology, University of New Mexico, Albuquerque, NM 87131 USA; 9 Stem Cell Institute, University of Minnesota, Minneapolis, MN 55455 USA; 10 Department of Cell Biology, Neurobiology and Anatomy, Medical College of Wisconsin, Milwaukee, WI 53226 USA

## Abstract

Schistosomiasis is a debilitating tropical disease caused by infection with parasitic blood flukes. Approximately 260 million people are infected worldwide, underscoring the clinical and socioeconomic impact of this chronic infection. Schistosomiasis is treated with the drug praziquantel (PZQ), which has proved the therapeutic mainstay for over three decades of clinical use. However, the molecular target(s) of PZQ remain undefined. Here we identify a molecular target for the antischistosomal eutomer — *(R)*-PZQ — which functions as a partial agonist of the human serotoninergic 5HT_2B_ receptor. *(R)*-PZQ modulation of serotoninergic signaling occurs over a concentration range sufficient to regulate vascular tone of the mesenteric blood vessels where the adult parasites reside within their host. These data establish *(R)*-PZQ as a G-protein-coupled receptor ligand and suggest that the efficacy of this clinically important anthelmintic is supported by a broad, cross species polypharmacology with PZQ modulating signaling events in both host and parasite.

## Abstract (web summary)

Schistosomiasis is caused by infection with the flatworm *Schistosoma*, and praziquantel is the drug of choice for its treatment. Here, Chan and colleagues identify praziquantel as a ligand for the human serotoninergic 5-HT_2B_ G-protein-coupled receptor, and reveal a function for praziquantel as a regulator of vascular tone in treated hosts.

## Introduction

Hundreds of millions of people are infected with parasitic worms^1^. One of the most burdensome infections is the neglected tropical disease schistosomiasis (Bilharzia) caused by parasitic flatworms of the genus *Schistosoma* ^2^. *Schistosoma* infections result from contact with fresh water containing cercariae, the free-swimming larval stage of the parasite. Cercariae penetrate the host’s skin, and then transform and mature into sexualized forms while undergoing a remarkable intravascular migration to defined vascular beds, where paired male and female worms complete their maturation and commence egg laying^2,3^. The debilitating impact of schistosomiasis results from the host’s immune response to schistosome eggs, deposited in prolific numbers in the liver, intestine and/or bladder where they elicit granulomatous inflammation, periportal fibrosis and hypertension^2^. Clinical outcomes span gastrointestinal and liver pathologies, genitourinary disease, anemia, malnutrition, and a heightened risk for comorbidities and HIV transmission. The associated disease burden encumbers third world economies with an annual loss of up to 70 million disability-adjusted life years^4,5^. Effective drug therapy for schistosomiasis is therefore a key healthcare priority.

Praziquantel, a tetracyclic tetrahydroisoquinoline derivative administered as a racemic mixture ((±)-PZQ), is the main drug therapy for combating schistosomiasis. PZQ causes rapid paralysis of schistosome musculature and subsequent tegumental damage that promotes immunological clearance of worms from the host. The World Health Organization estimates a considerable future demand for PZQ of 2 billion tablets over 5 years to support mass drug administration initiatives^5^. Clearly, the continued efficacy of PZQ is essential for this strategy and reports of PZQ-resistant worm isolates in both laboratory and field highlight an urgency in resolving how PZQ works^6–8^. Such knowledge would catalyze the development of next generation anthelmintics. Here we employ a variety of experimental approaches to demonstrate that the antischistosomal eutomer (*R*)-PZQ acts as a partial agonist of the human 5-HT_2B_ receptor, establishing (*R*)-PZQ as a GPCR ligand. These data will prioritize future screening of flatworm GPCRs for a *(R)-*PZQ target and underscore an activity of *(R)-*PZQ on 5-HT_2B_R signaling in the human host, manifest within the vascular beds where the adult parasites reside.

## Results

### Mammalian GPCR target(s) of PZQ

Prior work has demonstrated an ergomimetic like action of PZQ to engage flatworm bioaminergic signaling pathways^9^. Specifically, PZQ exposure caused a bipolar regeneration phenotype in regenerating free-living planarian flatworms that was phenocopied by ergot alkaloids acting as serotonergic antagonists, but opposed by 5-hydroxytryptamine (5-HT^9^). Similarly, in both adult schistosome worms (Supplementary Fig. 1) and schistosomules^9^, PZQ evoked a rapid paralysis counteracted by exogenous 5-HT. On the basis of these observations, we considered whether PZQ acts as a direct serotoninergic ligand to oppose 5-HT evoked G-protein-coupled receptor (GPCR) signaling.

**Fig. 1:**
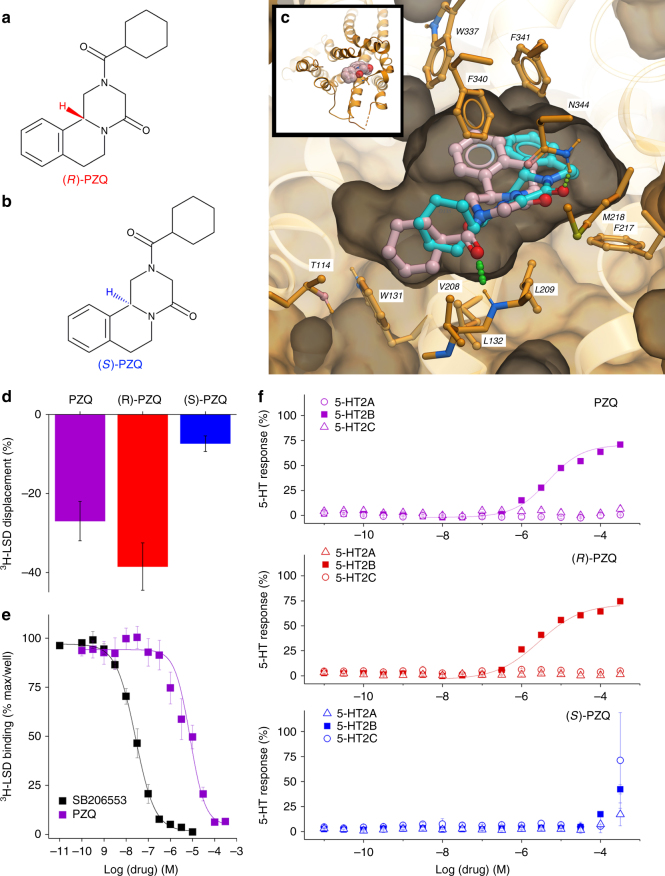
Resolution of stereoselective *(R)-*PZQ interaction with human 5-HT_2B_R. Structures of the PZQ enantiomers: active, **a** antischistocidal *(R)-*PZQ (top, red) and **b** *(S)-*PZQ (bottom, blue). **c** Docking poses of R-PZQ (light red) and S-PZQ (light blue) in the human 5HT_2B_R. Both isomers of PZQ dock favorably within the orthosteric pocket of the 5HT_2B_R crystal structure (Protein Databank Code: 5TVN). The R-isomer shows a better predicted binding score by 0.6 kcal/mole partially due to a more favorable hydrogen bond with the backbone NH group of L209. **d** Displacement of ^3^H-LSD binding at human 5-HT_2B_R by racemic PZQ ((±)-PZQ 10 µM, purple), *(R)-*PZQ (5 µM, red) and *(S)-*PZQ (5 µM, blue) in the PDSP primary screen (*n* = 5 for all measurements). **e** Complete binding displacement curves for ^3^H-LSD displacement at human 5-HT_2B_R for SB206553 (black) and (±)-PZQ (purple, *n* = 5 for both ligands). **f** Functional Assays. Ca^2+^ flux assays showing responses from fluo-4 loaded Flp-In T-REx cells stably expressing 5-HT_2A_R, 5-HT_2B_R, or 5-HT_2C_R at indicated concentrations of (±)-PZQ (purple, top), *(R)-*PZQ (red, middle) and *(S)-*PZQ (blue, bottom; *n* = 3 for each concentration) in cells expressing 5-HT_2A_R, 5-HT_2B_R, or 5-HT_2C_R

To investigate the possibility that PZQ engages serotoninergic GPCRs, in silico docking approaches were first applied. The resolved PZQ enantiomers—(*R*)-PZQ (the active, antischistocidal eutomer, Fig. 1a) and (S)-PZQ (Fig. 1b)—were screened against a panel of 3436 computational models, representing 1465 protein targets mostly of human origin, (‘MolScreen’). Both ligands were docked and scored into ensemble 4D^10^ models of various targets assembled in the Pocketome database^11^. The top candidate from the docking to pocket classification (‘dpc’ models) was the human 5-HT_2B_ receptor (5-HT_2B_R), with (*R*)-PZQ possessing the highest predicted affinity (Supplementary Table 1). Figure 1c depicts the orientation of both (*R*)-PZQ and (*S*)-PZQ within the 5-HT_2B_R orthosteric binding pocket from modeling simulations. Different binding orientations for both PZQ enantiomers were predicted with the (*R*)-isomer exhibiting a more favorable hydrogen bond with the backbone amine group of L209 in extracellular loop 2 of 5-HT_2B_R (Fig. 1c).

With these in silico predictions in hand, we profiled a broad panel of human GPCRs through the NIMH Psychoactive Drug Screening Program (PDSP^12^) first using racemic (±)-PZQ (10 µM) and then the resolved enantiomers (*(R)-*PZQ and *(S)-*PZQ^13^; Supplementary Fig. 2). (±)-PZQ exhibited a polypharmacological profile (Supplementary Fig. 3) consistent with predictions from the in silico modeling data (Fig. 1b). The ability of PZQ to engage host GPCRs likely explains prior reports of (±)-PZQ action on heart and smooth muscle^14,15^, and potentially other known activities of PZQ such as the bitterness of *(S)-*PZQ^16^, a taste transduced through GPCR signaling. This screen was then repeated using the individual enantiomers with the criterion for a positive screening hit defined as stereoselective inhibition of radioligand binding by *(R)-*PZQ, but not *(S)-*PZQ. This was observed at only one GPCR in the primary screen—the human 5-HT_2B_ receptor (5-HT_2B_R)—where inhibition seen with (±)-PZQ was attributable solely to *(R)-*PZQ (Fig. 1d, Supplementary Fig. 3). These data support the in silico modeling, although demonstrate more biological selectivity for *(R)-*PZQ over *(S)-*PZQ at 5-HT_2B_R.

PZQ interaction with 5-HT_2B_R was then validated using radioligand binding and functional assays. Analysis of [^3^H]-LSD displacement by (±)-PZQ revealed complete displacement of specific [^3^H]-LSD binding at 5-HT_2B_R by unlabeled (±)-PZQ (*K*_i_ = 5.3 µM, Fig. 1e). For functional activity, Ca^2+^ flux assays were performed in HEK293 cells expressing individual 5HT_2_R isoforms. *(R)-*PZQ evoked Ca^2+^ release in 5-HT_2B_R expressing cells at concentrations >1 µM (Fig. 1f). The peak amplitude of *(R)-*PZQ-evoked Ca^2+^ release was consistently less than evoked by 5-HT (Fig. 1d), suggesting action as a 5-HT_2B_R partial agonist. No Ca^2+^ release activity was observed using *(S)-*PZQ over a comparable concentration range (Fig. 1f). No Ca^2+^ release activity was observed in uninduced cells lacking 5-HT_2B_R expression, or cells expressing either 5-HT_2A_R or 5-HT_2C_R (Fig. 1f), indicating that *(R)-*PZQ activity was specific and selective for the 5-HT_2B_R. At other tested GPCRs, including several predicted by the modeling simulations (Supplementary Table 1), (±)-PZQ lacked Ca^2+^ release activity (Supplementary Fig. 4).

**Fig. 2:**
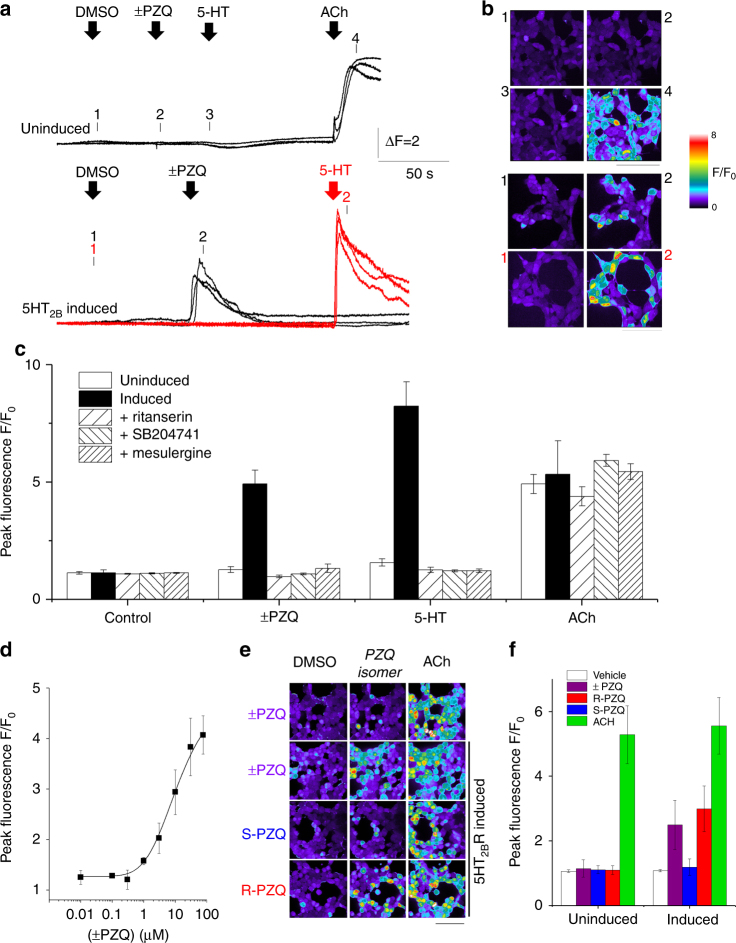
*(R)-*PZQ triggers Ca^2+^ release via 5-HT_2B_R. **a** Representative fluorescence traces from a single experiment where single cells loaded with fluo-4-AM in a HEK293 cell line prior to (top), or after (bottom) 5-HT_2B_R induction. Traces show fluorescence ratio following addition of indicated ligands (final concentrations (±)-PZQ (75 µM), 5-HT (100 nM) or ACh (100 µM)), or vehicle (DMSO, 0.05%). **b** Pseudocolored confocal images are displayed from the indicated times (‘1’–‘4’) on associated fluorescence traces as introduced in Fig. 1a. Scalebar, 80 µm. **c** Peak fluorescence ratio (F/F_0_, where ‘F’ represents fluorescence at peak and ‘F_0_‘ represents fluorescence at time = 0) from cumulative Ca^2+^ imaging after addition of vehicle, (±)-PZQ, 5-HT or ACh in uninduced (open bars) or induced cell lines (solid, hatched bars; *n* ≥ 3 independent inductions). Serotonergic antagonists (ritanserin (100 nM, final concentration), SB204741 (100 nM), or mesulergine (200 nM) were preincubated with induced cells prior to addition of (±)-PZQ (75 µM), 5-HT (10 nM), or ACh (100 µM). Data represent means ± s.e.m. of peak responses averaged from multiple cells (>20) from *n* ≥ 3 independent inductions. **d** Dose-response relationship for (±)-PZQ efficacy in cells induced for 5-HT_2B_R expression (*n* = 6 for each concentration). **e** Confocal Ca^2+^ imaging assays with (±)-PZQ and resolved enantiomers in uninduced (top row) and 5-HT_2B_R induced cell lines (bottom three rows). Experiments were performed with racemic PZQ ((±)-PZQ), and individual *(S)-*PZQ and *(R)-*PZQ stereoisomers (5 µM, final concentration). Scalebar, 80 µm. **f** Processed data set from experiments such as shown in **e** representing population mean ± s.e.m. (>20 cells) from *n* ≥ 3 independent inductions

Further characterization of PZQ-evoked Ca^2+^ release was performed in a 5-HT_2B_R inducible cell line by confocal imaging. Neither (±)-PZQ, nor 5-HT, evoked cellular Ca^2+^ transients in the absence of 5-HT_2B_R induction, whereas acetylcholine (ACh) elevated cytoplasmic Ca^2+^ through endogenous GPCRs (Fig. 2a, b). Following 5-HT_2B_R induction, both (±)-PZQ and 5-HT triggered Ca^2+^ signals (Fig. 2a, b). Prior incubation of induced cells with serotonergic blockers ritanserin, SB2047471, or mesulergine blocked PZQ and 5-HT evoked Ca^2+^ transients, but failed to impact ACh-evoked Ca^2+^ signals (Fig. 2c). Dose-response relationships revealed (±)-PZQ activity over the micromolar range (EC_50_ = 8.1 µM; Fig. 2d) with Ca^2+^ release activity solely caused by the *(R)-*enantiomer (Fig. 2e, f). The 5-HT_2B_R blocker LY272015 also inhibited PZQ-evoked Ca^2+^ signals (Supplementary Fig. 5).

Next, we examined PZQ-evoked signaling using a transcriptional reporter assay, designed to monitor Ca^2+^-dependent NFAT translocation (Supplementary Fig. 6A). In HEK293 cells expressing the reporter construct alone, neither (±)-PZQ nor 5-HT increased the basal luminescence signal (Supplementary Fig. 6B). In cells transiently transfected with 5-HT_2B_R, both PZQ and 5-HT elicited NFAT translocation and this response was blocked by ritanserin (Supplementary Fig. 6B). *(R)-*PZQ activation of NFAT occurred over the micromolar range (Supplementary Fig. 6C), again less potent and penetrant than observed with 5-HT (Figs. 1d, 2d). Therefore, multiple experimental approaches demonstrated *(R)-*PZQ acted as a partial agonist at human 5-HT_2B_R.

### Action of (R)-PZQ at host GPCRs

**Fig. 3:**
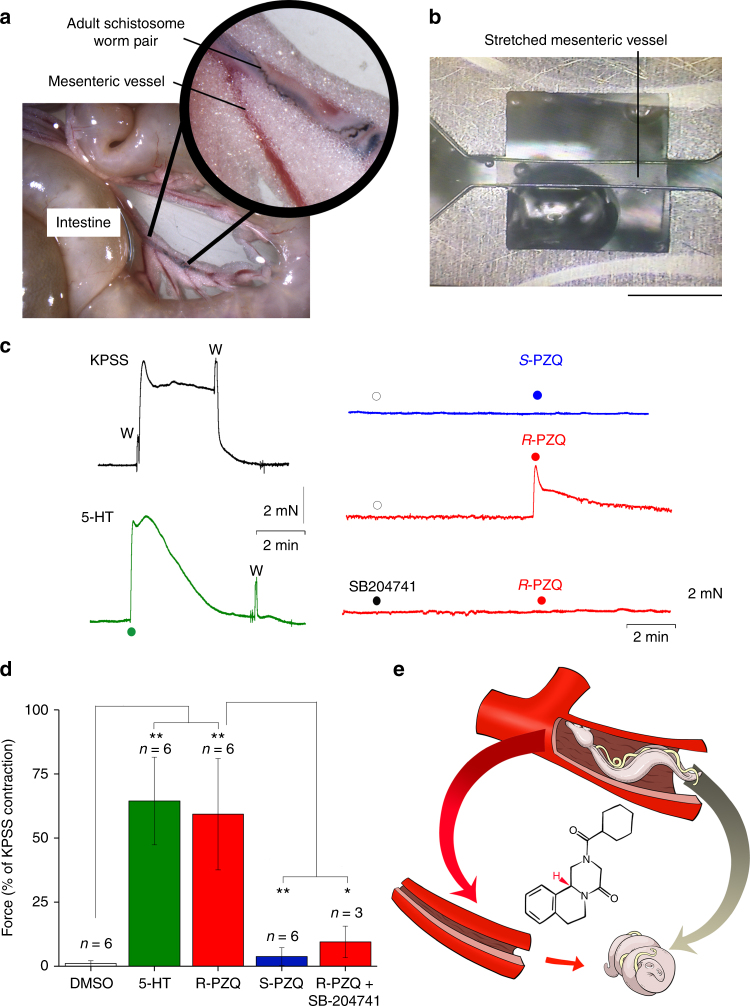
*(R)-*PZQ constricts mouse mesenteric vasculature. **a** Image of mesentery surrounding mouse intestine, showing blood vessel containing a schistosome worm-pair (inset). **b** Representative image of a surgically isolated piece of mouse mesenteric artery held under tension between two clamped wires. Scalebar, 1 mm. **c** Changes in tension triggered by varied manipulations. Left, sustained contraction of vessels in KPSS and 5-HT (10 µM). Right, tension in individual arteries incubated in the presence of vehicle (DMSO, open circle) or SB204741 (10 µM, closed circle), and challenged with *(R)-*PZQ (50 µM) or *(S)-*PZQ (50 µM). W, wash; complete media exchange. Preincubation of strips with SB204741 (10 µM) attenuated *(R)-*PZQ evoked vasoconstriction. **d** Cumulative data set from experiments reporting effects of ligands relative to KPS(S)-evoked contractile tension, concentrations of ligands: 5-HT (1 µM), *(R)-*PZQ (50 µM), *(S)-*PZQ (50 µM), SB204741 (10 µM). Replicate number for each measurement as indicated, where individual measurements reflect the response from a naïve artery isolated from different mice. Data are presented as mean ± s.d. Probability, *p* < 0.05 (*), *p* < 0.01 (**). **e** Schematic model (not to scale) depicting *(R)-*PZQ action on both parasite (contraction) and host vasculature (contraction, increased perfusion pressure) facilitating hepatic shift of worms

These data are significant for two reasons. First they identify PZQ as a *bona fide* GPCR ligand at a defined molecular target. This discovery prioritizes future screening of flatworm GPCRs (~120 GPCRs in schistosomes^17^) for a receptor selectively engaged by *(R)-*PZQ that could be a key target for future antischistosomal drug development^18,19^. Second, these data suggest PZQ—conventionally viewed as a selective antiparasitic therapy—can interact with endogenous 5-HT_2B_Rs in the human host. One pathophysiologically relevant effect would be an activity of *(R)-*PZQ on host mesenteric vascular beds, a destination of the mature, paired adult *S. mansoni* and *S. japonicum* blood flukes and sites where egg laying commences^2,3^ (Fig. 3a). After drug treatment, worms are rapidly displaced from their mesenteric habitat to the liver where elimination occurs. This ‘hepatic shift’ is a frequently used assay for drug efficacy and has been attributed to loss of worm muscle tone evoked by antischistosomal agents^20,21^ as seen in vitro (Supplementary Movie 1). Serotonergic ligands are well known regulators of the tone of arteries and veins^22,23^, including mesenteric vessels^23^, such that PZQ-evoked changes in mesenteric blood flow may help ‘flush’ PZQ-paralyzed worms toward the liver. Levels of PZQ within the splanchnic vasculature likely reach levels an order of magnitude higher than peak plasma concentrations that are measured after first-pass metabolism of the drug (>4 µM for 40 mg/kg human dosing, even higher in mouse models^24,25^). Therefore, PZQ concentration within mesenteric vessels falls well within the concentration range for causing host serotonergic effects demonstrated in vitro (Figs. 1, 2).

The activity of PZQ within the mesenteric vasculature of uninfected mice was assessed by measuring mesenteric artery tone using wire myography. Mounted vessel segments (Fig. 3b) exhibited a rapid, sustained contraction to high K^+^ media which reversed upon media exchange (Fig. 3c). Addition of 5-HT to resting vessel segments also elicited a protracted, contractile response that declined over time (Fig. 3c). Addition of *(R)-*PZQ to naive vessels mimicked the profile of 5-HT evoked contractions (Fig. 3c), and this effect was prevented by preincubation with SB204741, a 5-HT_2B_R antagonist (Fig. 3c). No changes in resting tone were observed with *(S)-*PZQ. PZQ also evoked contraction of hepatic portal vein segments (Supplementary Fig. 7), again mimicking 5-HT action^26^. The cumulative data set demonstrates *(R)-*PZQ caused mesenteric artery vasoconstriction, mimicking the action of 5-HT (Fig. 3d).

In conclusion, we demonstrate that PZQ is a GPCR ligand, with antischistocidal *(R)-*PZQ acting as a low micromolar affinity partial agonist at human 5-HT_2B_ receptors. Activity at both parasite and host receptors—both targets potentially being GPCRs—likely contributes to clinical efficacy of PZQ by combining a deleterious paralytic effect on the parasite (Supplementary Movie 1) with beneficial host effects that promote worm clearance. We propose PZQ causes contraction of both the parasite and host mesenteric vessels to increase perfusion pressure and flush paralyzed worms to the liver (Fig. 3e). PZQ activity at host 5-HT_2B_Rs may be further enhanced in individuals infected with chronic schistosomiasis where pathological changes slow drug elimination^24^, heighten serotonergic sensitivity^26^ and lower basal arteriolar resistance^27^. Further, the beneficial effects of host GPCR engagement may extend beyond the vasculature given a role for 5HT_2B_Rs in regulating inflammation, liver fibrosis and regeneration: all outcomes relevant to chronic schistosomiasis pathology^28,29^. In conclusion, these data support a strategy for developing novel anthelmintics with cross species polypharmacology to reinforce disease mitigating actions in both host and parasite.

## Methods

### Reagents

Ritanserin, SB204741 and mesulergine were from Tocris Bioscience. All other ligands were from Sigma-Aldrich. Cell culture reagents were from Invitrogen. PZQ enantiomers ((*R*)-[-]PZQ and (*S*)-[+]PZQ) were resolved (Supplementary Fig. 2) using methods published by Woelfle et al.^13^.

### Mammalian GPCR profiling

PZQ (racemic and enantiomers) were screened against human GPCRs using primary and secondary assays coordinated through the NIMH Psychoactive Drug Screening Program (PDSP). Full details describing these methods are available in the PDSP Assay Protocol Book available online (https://pdspdb.unc.edu/pdspWeb/). PZQ was dissolved in 100% ethanol at a concentration of 100 mM to achieve a maximal assay concentration (up to 100 µM) for both binding and functional assays. Ca^2+^ flux experiments utilized tetracycline-inducible stable cell lines for 5HT_2A_, 5HT_2B_, and 5HT_2C_ receptors initially generated using the Flp-In 293 T-REx tetracycline inducible system^30^ (a gift from the Roth Lab, Invitrogen). Cells were maintained in DMEM (ThermoFisher, 10569010) supplemented with 10% dialyzed fetal bovine serum (Gibco, 26400044), 50 U/mL Penicillin-Streptomycin (ThermoFisher Scientific, 15140122), 10 µg/ml Blasticidin (Research Products International, B12150-0.1) and 100 µg/ml Hygromycin B (ThermoFisher Scientific, 10687010) at 37 °C and 5% CO_2_. For Ca^2+^ flux assays, receptor expression was induced with 2 µg/mL tetracycline, and cells were plated into white 384 clear bottom, tissue culture plates in 40 μL of DMEM containing 1% dialyzed FBS at a density of ~15,000 cells per well the day before the assay. Next day, media was decanted and 20 µL per well of Fluo-4 Direct dye (Invitrogen) was added and incubated for 1 h at 37 °C. The cells were stimulated with test compounds diluted in drug buffer (HBSS, 20 mM HEPES, 0.1% BSA, 0.01% ascorbic acid, pH 7.4) and calcium flux was measured using a FLIPR^TETRA^ (Molecular Devices). Plates were read for fluorescence initially for 10 s (1 read per second) to establish a baseline, and then stimulated with drug dilutions or buffer and read for an additional 110 s. Peak fluorescence in each well was normalized to maximum-fold increase over baseline. The data were normalized to maximum peak fold over basal fluorescence by 5-HT (100%) and baseline fluorescence (0%). The data were analyzed using the sigmoidal dose-response function built into GraphPad Prism 5.0.

### In silico modeling

(*R*)-PZQ and (*S*)-PZQ were screened against a panel of 3436 computational models designed to predict activities of arbitrary chemical compounds, representing 1465 proteins targets mostly of human origin (so called ‘MolScreen’ panel). The earlier version of this set of models that included different classes of targets^31^ described and validated the panel. Two main types of three dimensional docking models were used: ‘dpc’ models (349 models representing the conformational ensembles of binding pocket conformations from the Pocketome database^11^) and ‘dfa’ models (pharmacophoric field models based on the continuous three dimensional pharmacophoric fields of the diverse co-crystallized ligands from the Pocketome database, enhanced by training on the activity data from the ChEmbl database^32,33^).

### Confocal Ca^2+^ imaging

For single-cell confocal Ca^2+^ imaging, the 5HT_2B_ inducible cell line was seeded onto collagen-coated, 35 mm glass bottom dishes (MatTek, P35GCOL-0-14-C) at a density of 1 × 10^4^ cells three days prior to imaging. Two days prior to imaging, 5-HT receptor expression was induced by supplementing the growth media with 1 µg/ml doxycycline (Sigma-Aldrich, D3447). Two hours prior to conducting experiments, growth medium was exchanged for EMEM supplemented with 1% dialyzed FBS, plus antibiotics. The cells were washed twice with Hank’s balanced salt solution (HBSS), and incubated with Fluo-4-AM (4 µM) and Pluronic F127 (0.4%) for 25 minutes at room temperature. The cells were then washed twice with HBSS, and left at room temperature (30 min) for de-esterification. Dishes were mounted on an Olympus IX81 microscope and fluorescence changes (*λ*_ex_ = 488 nM, (*λ*_em_ = 513 ± 15 nm bandpass) monitored using a Yogokawa spinning disk confocal (CSU-X-M1N) and an Andor iXon Ultra 888 EMCCD camera. HEK293 cell lines were sourced from ATCC (CRL-1573) and found to be negative for mycoplasma contamination.

### Wire myography

Swiss Webster mice (female, aged 10–13 weeks) were obtained from Charles River Laboratories. Measurements of mouse mesenteric vessel tone were made using a multimyograph system (DMT, Aarhus, Denmark). Vessel strips were isolated from second order mesenteries and equilibrated for ≥30 min in gassed (95% O_2_, 5% CO_2_), physiological saline solution (PSS, 130 mM NaCl, 4.7 mM KCl, 1.18 mM KH_2_PO_4_, 1.17 mM MgSO_4_, 14.9 mM NaHCO_3_, 5.5 mM dextrose, 0.026 mM EDTA, 1.6 mM CaCl_2_, pH 7.4 at 37 °C). To identify the optimal pre-stretch value for experiments, a normalization factor (IC_1_/IC_100_) was calculated for individual test strips^34,35^, defined as the ratio of the internal circumference at which the maximum response to vasoconstriction (KCl, plus 40 µM norepinephrine) was observed (IC_1_), divided by the internal circumference at which a transmural wall pressure of 100 mm of Hg is attained on a length-tension plot overlayed with a La Place transformation isobar (IC_100_). After vessel equilibration, reactivity was measured under isometric conditions in response to KCl (KPSS, 74.7 mM NaCl, 60 mM KCl, 1.18 mM KH_2_PO_4_, 1.17 mM MgSO_4_, 14.9 mM NaHCO_3_, 5.5 mM dextrose, 0.026 mM EDTA, 1.6 mM CaCl_2_, pH 7.4 at 37 °C) or indicated ligands, as per published protocols. All animal experiments followed ethical regulations approved by the University of Minnesota IACUC committee and reviewed by the National Institutes of Health (NIAID).

### Adult *Schistosoma mansoni* mobility assays

Infected mice were provided by the Biomedical Research Institute (33) from which adult worms were collected by portal perfusion 6–8-week-post infection. Mobility experiments were conducted 24–48 h after worm collection using a compound microscope equipped with a digital video camera to acquire video recordings (3 frames per second for 1–2 min) of worms exposed to various drugs. Analysis was performed in ImageJ after file import using the Bio-Formats plugin. Differences in illumination were corrected using the stack deflicker function of the wrMTrck plugin. Images were processed by converting to binary format, and mobility was assayed by measuring the average difference in pixels resulting from subtracting two consecutive frames, providing a measurement of the worms displacement over that period (~0.3 s). This calculation was performed for each frame in the video, and the results were averaged over the length of the recording to provide a metric of worm movement. Unless otherwise noted, values reported represent the mean ± standard error of at least three independent experiments.

### Mammalian GPCR profiling

Primary GPCR screening assays were coordinated through the NIMH Psychoactive Drug Screening Program (PDSP). Methods and statistical analyzes for radioligand binding and functional assays are available in the PDSP Assay Protocol Book available online (https://pdspdb.unc.edu/pdspWeb/). Ca^2+^ flux assays were performed as similarly described, except experiments utilized muscarinic M1, M3, M5 receptors stably expressed in CHO cells. For experiments measuring cAMP, experiments utilized co-transfection with the cAMP split-luciferase reporter GloSenso(R)-22F plasmid (Promega, 1:1 ratio with receptor) and were read for luminescence on a TriLux Microbeta (Perkin Elmer). For Gi/o mediated cAMP inhibition, forskolin (1 µM) was used to stimulate cAMP via adenylyl cyclase activation.

### NFAT reporter assay

The Ca^2+^ sensitive transcriptional luciferase reporter pGL4.30[luc2P/NFAT-RE/Hygro] (Promega, E8481) was transiently transfected into the 5HT_2B_R stable cell line. Briefly, 3 × 10^6^ cells were plated in a Nunc Cell Culture Treated flask (25 cm^2^, ThermoFisher) and transfected with lipofectamine 2000 (ThermoFisher) plus 1 µg of plasmid DNA according to the manufacturer’s protocol. The following day, culture media was replaced with induction media (DMEM + 10% dialyzed FBS supplemented with 1 µg/ml doxycycline), and 24 h later cells were re-plated into 96 well, solid white plates (Costar, 3917). After allowing 3 h for cells to adhere, drugs were added at 20× concentration. For antagonist experiments, cells were incubated with 5HT_2B_ R antagonists for 2 h prior to subsequent addition of agonist. After 18 h culture in the presence of agonist, plates were assayed using the ONE-Glo Luciferase Assay System (Promega, E6120) and read on a GloMax-Multi Detection System plate reader (Promega).

### Statistical analysis

Except for the myography experiments, data (PDSP data, confocal calcium imaging) were analyzed using the two tailed Student’s *t*-test, and are reported except where explicitly indicated as mean ± s.e.m for independent biological replicates (defined as independent transfections or inductions). For myography experiments, where the amounts of resolved enantiomers were limiting, measurements were made in vessels isolated from 3–6 mice, a sample size providing a α-significance criterion of 0.05 and a β of 0.9. No randomization or blinding was used in the animal studies, as no comparative analyzes were involved. The statistical significance between experimental groups in myography experiments was determined using the Mann–Whitney test and data are presented as mean ± s.d. for these assays. Replicate numbers for individual experiments are outlined within Figure Legends or Figures as appropriate for clarity. Probability values of *p* < 0.05 were considered statistically significant.

### Data availability

The authors declare that the data supporting the findings of this study are available within the paper and its Supplementary Data Files or from the corresponding author on reasonable request.

## Electronic supplementary material

Supplementary Information

Description of Additional Supplementary Files

Supplementary Movie 1
