# Supplementary material for: The anthelmintic praziquantel is a human serotoninergic G-protein-coupled receptor ligand
Source: Nat Commun. 2017 Dec 5;8:1910. doi: 10.1038/s41467-017-02084-0 (PMC5716991; doi:10.1038/s41467-017-02084-0)
Supplement: Supplementary file 1 — Supplementary Information [file 41467_2017_2084_MOESM1_ESM.pdf]

Supplementary Figure 1

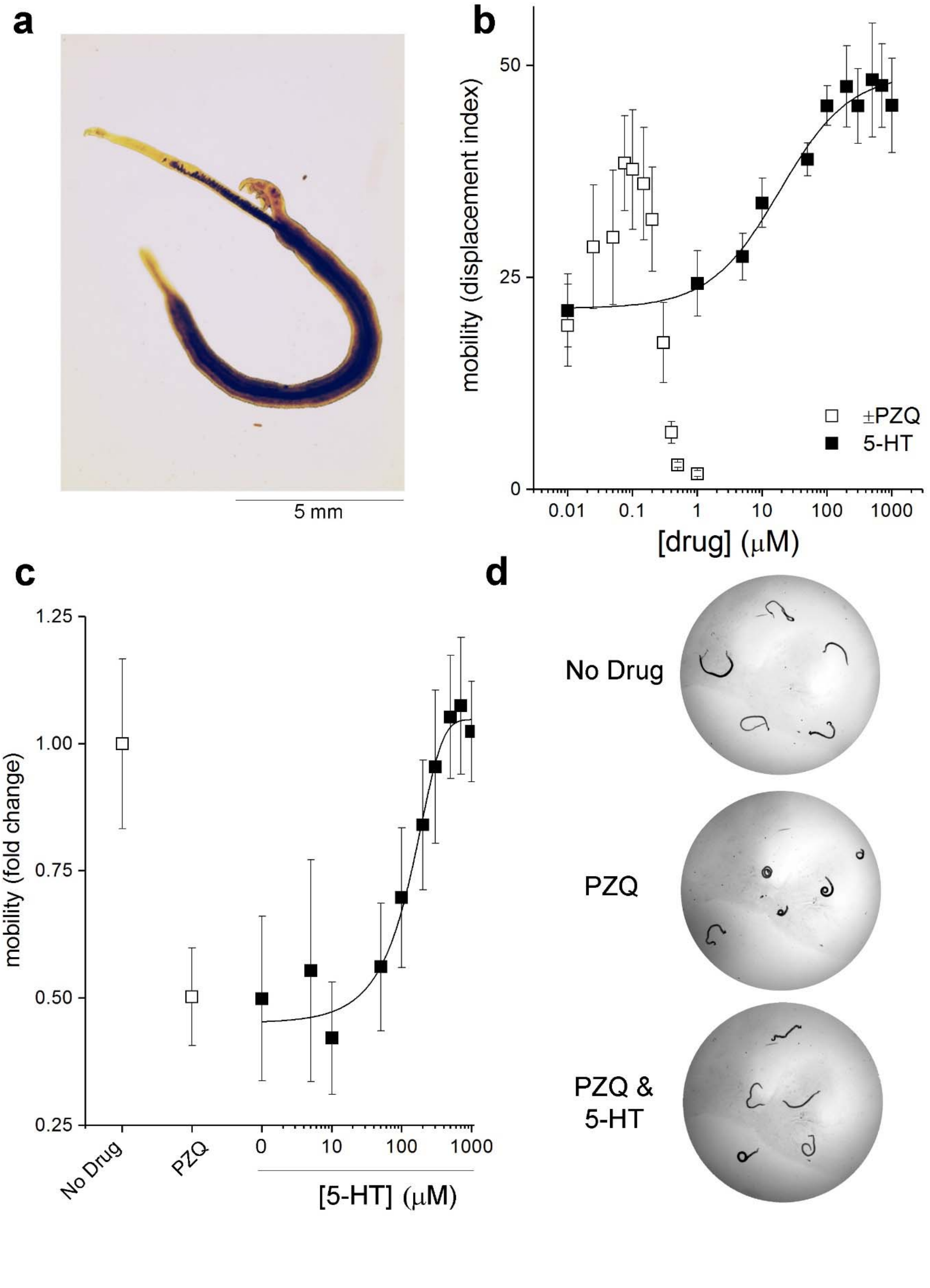

Supplementary Figure 1. **PZQ is a potent, biphasic modulator of schistosome musculature.** (**a**) Representative image of a paired pair of parasitic *S. mansoni* adult worms, with the female residing within the ventral groove of the male. (**b**) Dose-response curves for female *S. mansoni* worms exposed to the indicated concentrations of PZQ (open squares) or 5-HT (closed squares). Low concentrations of PZQ stimulate movement while higher concentrations effect a rapid paralysis. 5-HT is myoexcitatory causing stimulation of movement. Data represent mean±s.d. for n=4 independent assays. (**c**) 5-HT overcomes (±)-PZQ-evoked paralysis. Mobility of female worms normalized in the absence of any drug, following (±)-PZQ exposure (0.3μM, open squares) and on supplementation with the indicated concentrations of 5-HT (solid squares). (**d**) Representative images from videos used for quantification of mobility in (c).

Supplementary Figure 2

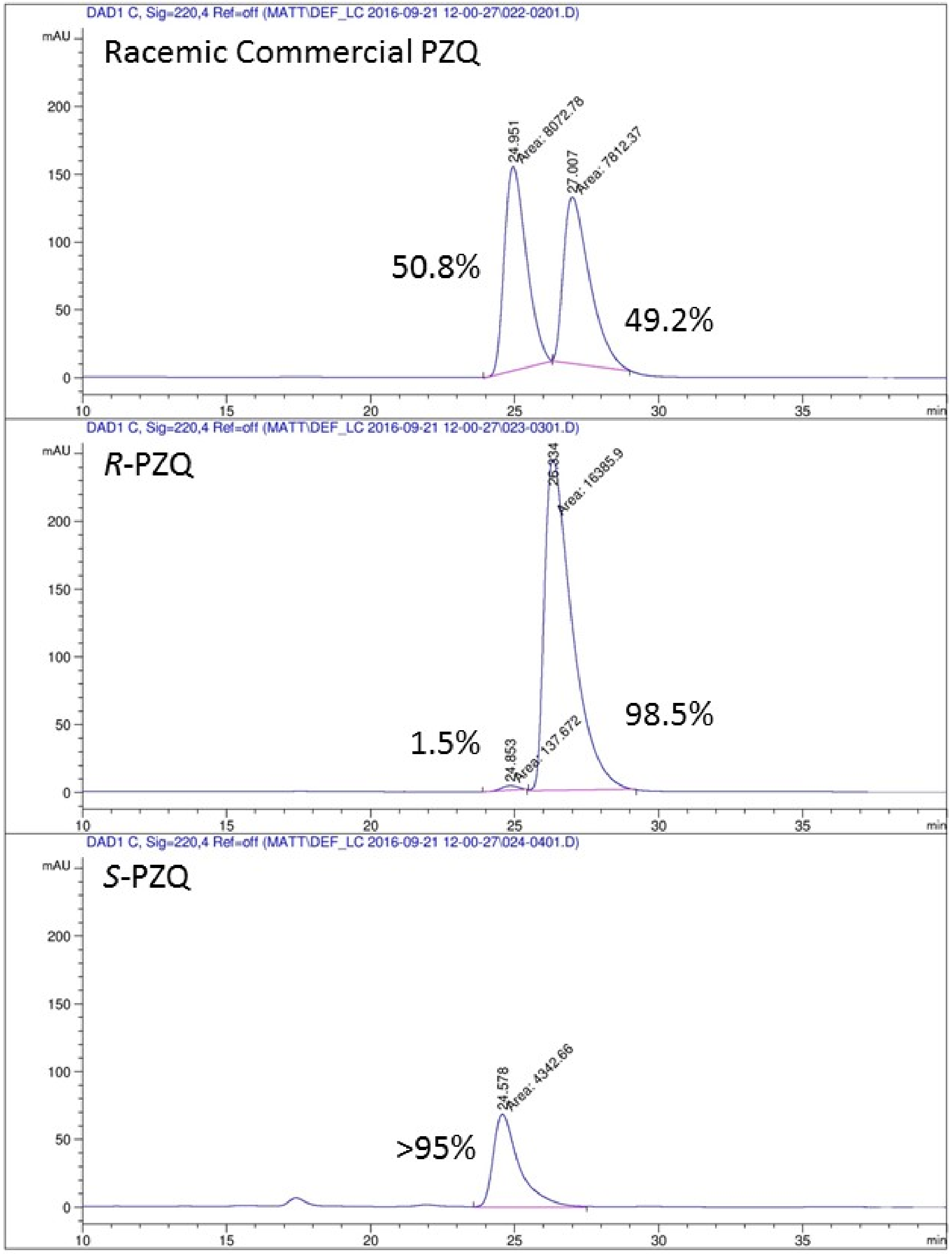

Supplementary Figure 2. Chiral HPLC analysis of racemic PZQ ((±)-PZQ, top), *(R)*-PZQ (middle), and *(S)*-PZQ samples. The *(R)*-PZQ sample contained a small amount of the *(S)*-enantiomer (1.5%). The *(S)*-PZQ sample contained no identifiable *(R)*-enantiomer, however the presence of a small amount (<5%) cannot be ruled out due to imperfect separation between the two enantiomers. Conditions: Chiralpak AD-H column, 150mm x 4.6mm x 5µm, 90/10 hexane/IPA, 0.7 mL/min.

Supplementary Figure 3

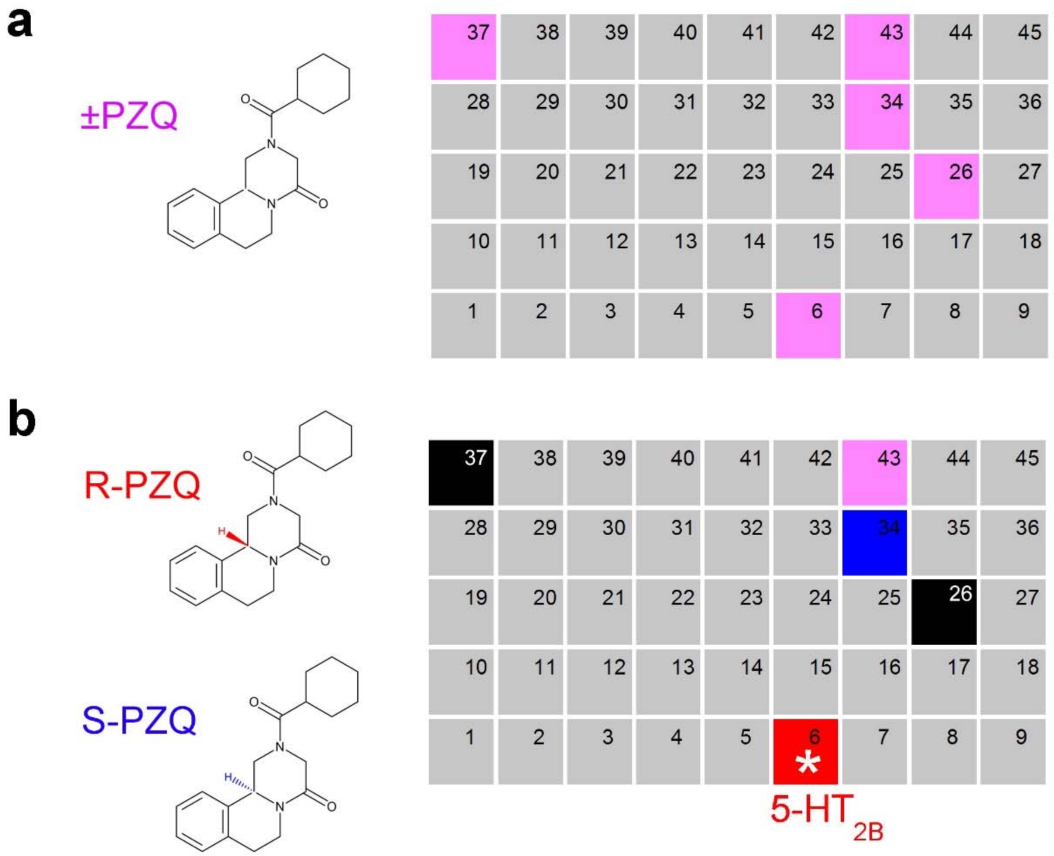

Supplementary Figure 3. **Primary screen of (±)-PZQ, *(R)*-PZQ and *(S)*-**PZQ **against human GPCR panel.** (**a**) PDSP primary screen with racemic (±)-PZQ (left). Heat map depicts summary of where pink coloration depicts >20% inhibition of radioligand binding by PZQ (10μM) in each of 4 independent biological assays, with 3 well replicates per individual run. Five provisional hits were 5-HT$_{2B}$ (number 6), D$_5$ (26), KOR (34), M$_3$ (37) and SERT (43). Full target key for PDSP screen, 1=5-HT$_{1A}$, 2=5-HT$_{1B}$, 3=5-HT$_{1D}$, 4=5-HT$_{1E}$, 5=5-HT$_{2A}$, 6=5-HT$_{2B}$, 7=5-HT$_{2c}$, 8=5-HT$_3$, 9=5-HT$_{5A}$, 10=5-HT$_6$, 11=5-HT$_7$, 12=α_{1A}, 13=α_{1B}, 14=α_{1D}, 15=α_{2A}, 16=α_{2B}, 17=α_{2C}, 18=β_1, 19=β_2, 20=β_3, 21=BZP rat brain site, 22=D$_1$, 23=D$_2$, 24=D$_3$, 25=D$_4$, 26=D$_5$, 27=DAT, 28=DOR, 29=GABA$_A$, 30=H$_1$, 31=H$_2$, 32=H$_3$, 33=H$_4$, 34=KOR, 35=M$_1$, 36=M$_2$, 37=M$_3$, 38=M$_4$, 39=M$_5$, 40=MOR, 41=NET, 42=PBR, 43=SERT, 44=Σ_1, 45=Σ_2. (**b**) repeat of primary screen with resolved enantiomers: *(R)*-PZQ (red) and *(S)*-PZQ (blue). Effects at D$_5$ and M$_3$ (black, #26 & #37) were not reproduced in binding or functional assays (Supplementary Figure 4). No stereochemical selectivity was seen for inhibition of SERT (pink, #43), whereas inhibition at 5-HT$_{2B}$ was selective for the schistocidal *(R)*-PZQ (red, #6) whereas KOR inhibition was attributable to *(S)*-PZQ (blue, #34). 5-H$_{T2B}$ represented the only screened GPCR where inhibition seen with racemic PZQ ((±)-PZQ) was attributable solely to the activity of the antischistocidal eutomer, *(R)*-PZQ.

Supplementary Figure 4

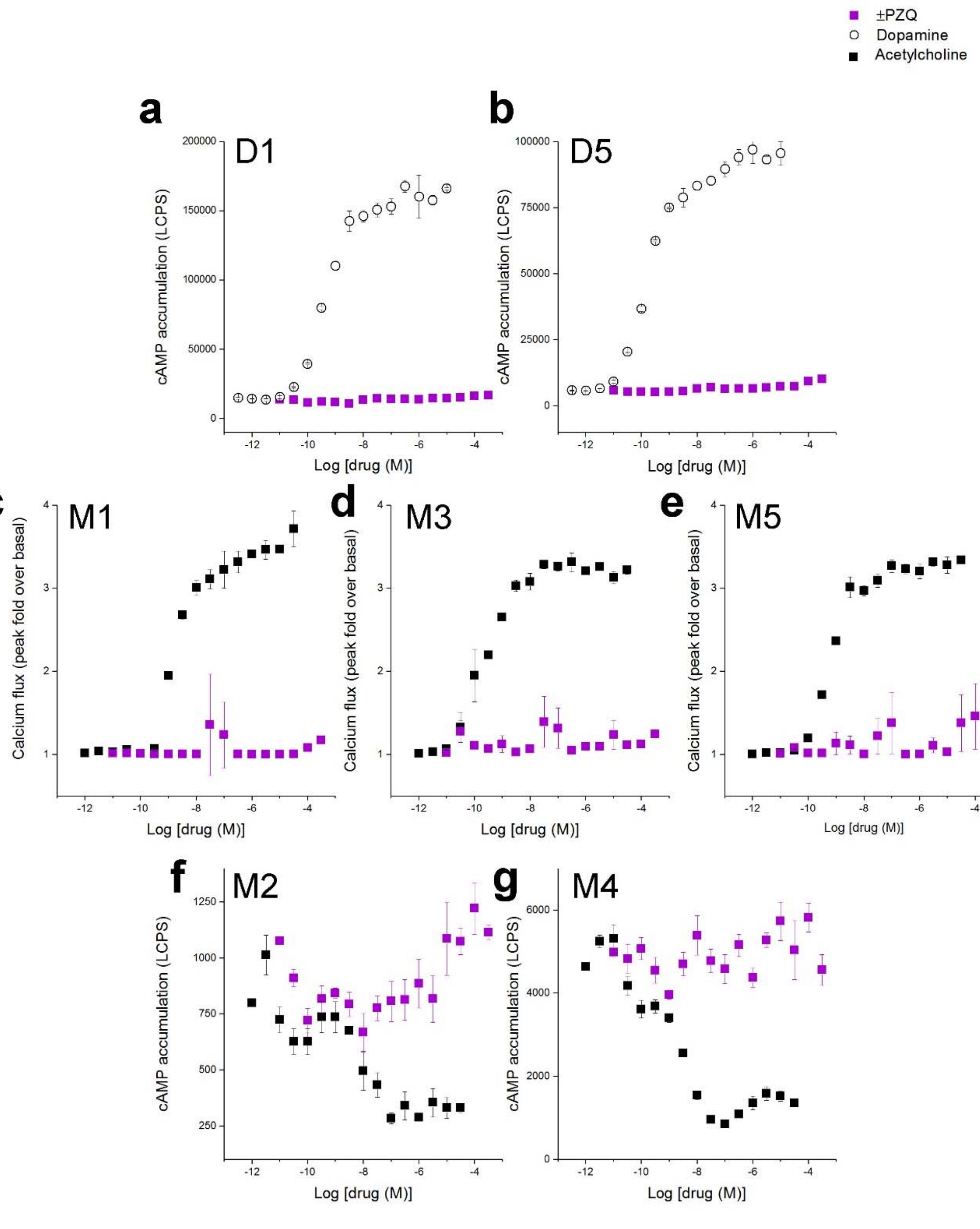

Supplementary Figure 4. **Lack of PZQ efficacy at other families of GPCRs**. PZQ was assessed for activity at human dopaminergic GPCRs (D_1, D_5; G_s coupled, top) and muscarinic GPCRs: M_1, M_3 and M5 (G_q coupled, middle), M_2 and M_4 (G_i coupled, bottom) using plate reader assays. Data are from a representative experiment mean±s.d. from multiple wells.

Supplementary Figure 5

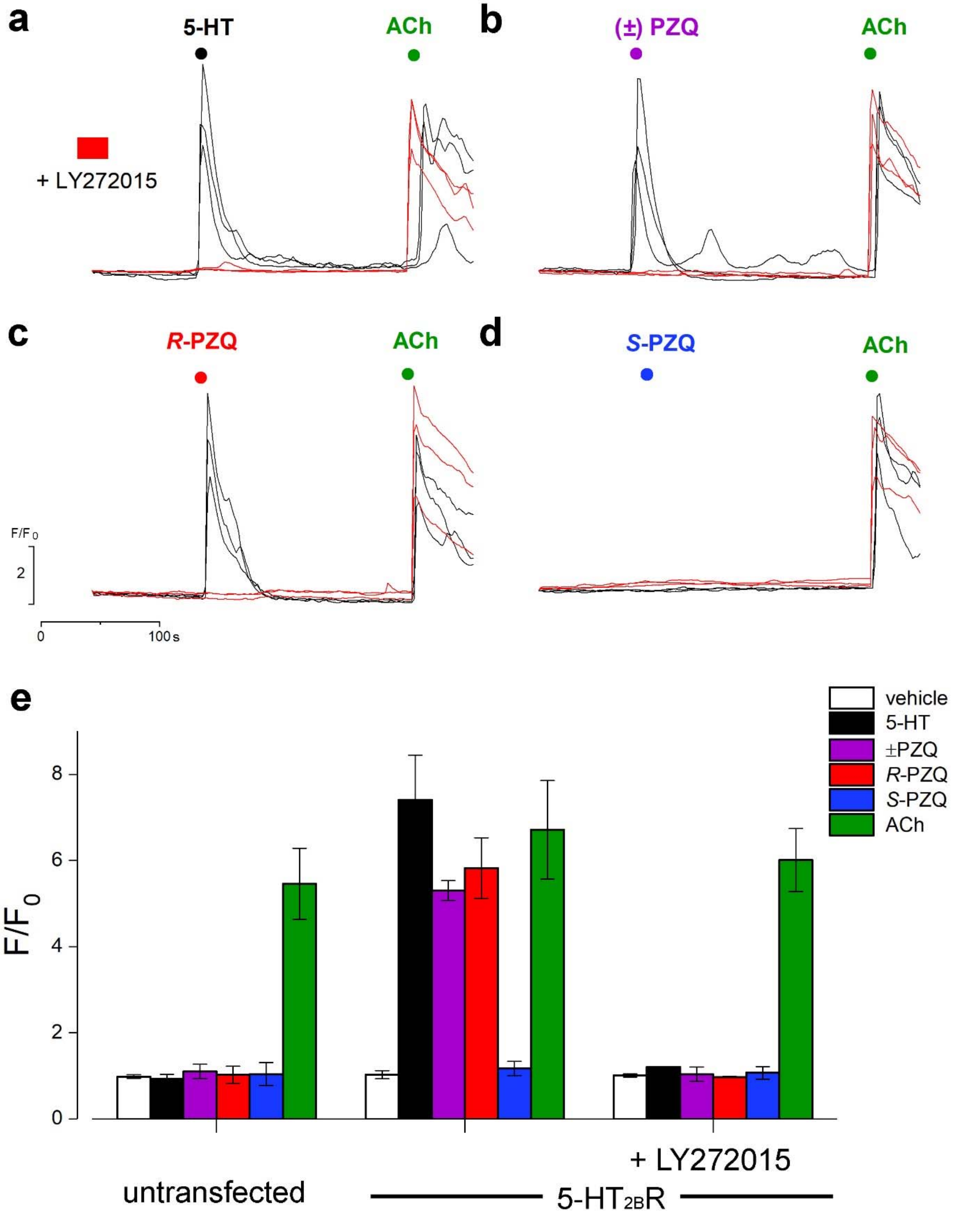

Supplementary Figure 5. **LY272015 blocks PZQ-evoked Ca^{2+} responses**. The effectiveness of the 5-HT_{2B}R blocker, LY272015, was assessed in HEK-293 cells transiently transfected with 5-HT_{2B}R. Representative traces of Ca^{2+} responses from single cells elicited by (**a**) 5-HT (100nM), (**b**) (±)PZQ (10μM), (**c**) *(R)*-PZQ (5μM) and (**d**) *(S)*-PZQ (5μM) before or after (red traces) treatment of cells with LY272015 (10min pretreatment with 100nM LY272015). (**e**) Cumulative dataset from these experiments. Data represent mean±s.e.m. of peak responses from multiple cells (>20) averaged from n≥3 independent transfections.

Supplementary Figure 6

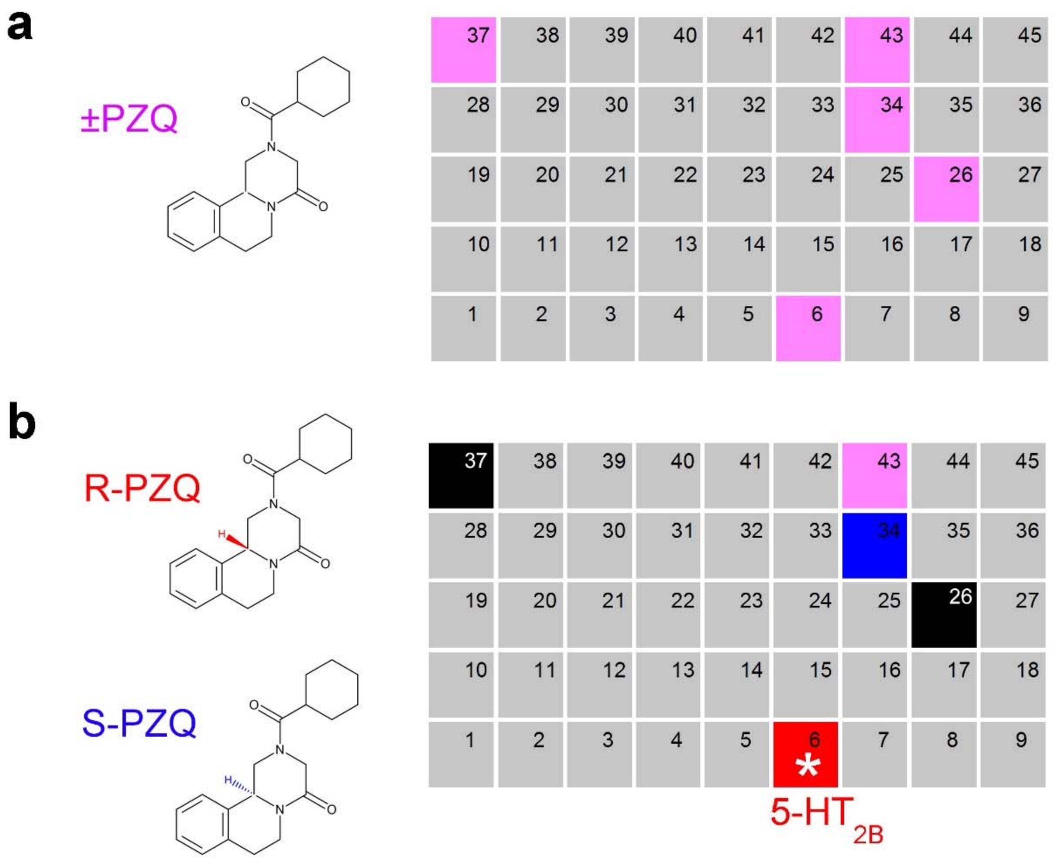

Supplementary Figure 6. PZQ triggers NFAT translocation via stereoselective engagement of 5-HT$_{2B}$R. (**a**) Schematic of NFAT-reporter assay. Activation of GPCRs coupled to G_q initiates a Ca^{2+}dependent NFAT translocation and activation of a NFAT-dependent reporter. (**b**) Comparison of luminescence signals in control (open bars) and cells transiently transfected with 5-HT$_{2B}$R cDNA (solid bars) in response to indicated ligands ((±)-PZQ [50µM], 5-HT [100nM], (±)-PZQ and ritanserin [100nM]) compared to the control signal (ionomycin [1µM] + PMA [16nM]). (**c**) Dose-response relationship of enhancement of luminescence values (fold-change versus basal) for indicated concentrations of 5-HT, (±)-PZQ, or the individual stereoisomers *(R)*-PZQ (red) and *(S)*-PZQ (blue). Measurements were made 18hrs after exposure to drugs. Data represent mean±s.d. from a representative assay performed in parallel with each of the ligands.

Supplementary Figure 7

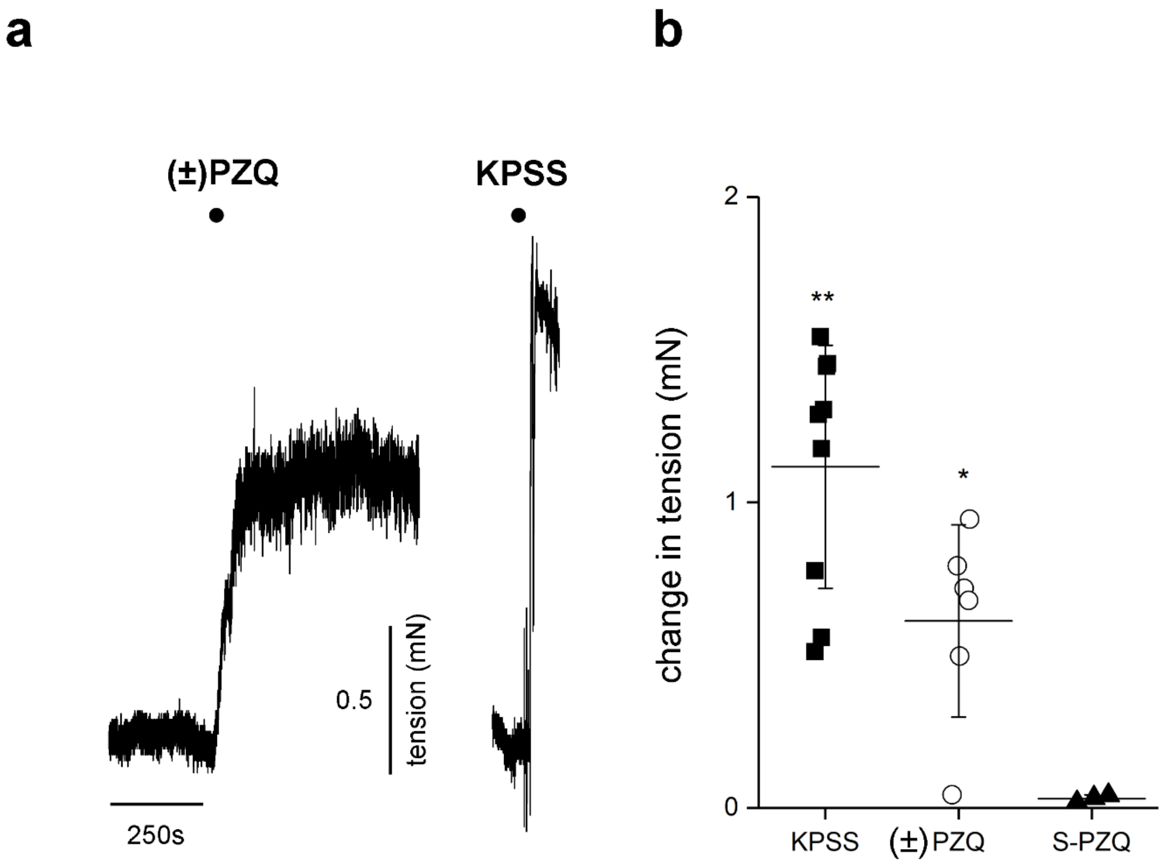

Supplementary Figure 7. PZQ contracts hepatic portal vein. (**a**) Representative myography traces from a segment of mouse hepatic portal vein, showing changes in tension evoked by (±)-PZQ (left, 100µM) and KPSS (right). (**b**) Comparison of peak tension elicited by indicated drug treatments. Individual measurements, mean±s.d for KPSS (solid squares), (±)-PZQ (open circles, 100µM) and S-PZQ (solid triangles, 50µM) are shown. Statistical significance was analysed using the Mann-Whitney test, probability $p<0.01$ (**) or $p<0.05$ (*) versus *S*-PZQ.

Target	Code	pPvalue	Mol_pKd	pKd_Error	Drug
5-hydroxytryptamine receptor 2B	5HT2B	1.99	6.99	1.44	(*R*)-PZQ
5-hydroxytryptamine receptor 2B	5HT2B	1.91	6.89	1.44	(*S*)-PZQ
Muscarinic acetylcholine receptor M2	ACM2	1.48	6.33	1.24	(*R*)-PZQ
Muscarinic acetylcholine receptor M3	ACM3	1.18	5.88	1.20	(*R*)-PZQ
Kappa-type opioid receptor	OPRK	1.13	5.81	1.28	(*S*)-PZQ
D(3) dopamine receptor	DRD3	1.12	5.80	1.28	(*R*)-PZQ
Muscarinic acetylcholine receptor M3	ACM3	0.81	5.26	1.20	(*S*)-PZQ
Kappa-type opioid receptor	OPRK	0.79	5.22	1.28	(*R*)-PZQ
Delta-type opioid receptor	OPRD	0.78	5.20	1.23	(*S*)-PZQ
Muscarinic acetylcholine receptor M2	ACM2	0.76	5.16	1.24	(*S*)-PZQ
Adenosine receptor A2a	AA2AR	0.45	4.25	1.26	(*R*)-PZQ
Adenosine receptor A2a	AA2AR	0.48	3.54	1.26	(*S*)-PZQ
Delta-type opioid receptor	OPRD	0.39	3.44	1.23	(*R*)-PZQ
Beta-2 adrenergic receptor	ADRB2	0.82	2.41	1.27	(*R*)-PZQ
Beta-2 adrenergic receptor	ADRB2	0.23	ND	ND	(*S*)-PZQ
D(3) dopamine receptor	DRD3	0.05	ND	ND	(*S*)-PZQ
Corticotropin-releasing factor receptor 1	CRFR1	0.55	ND	ND	(*S*)-PZQ
Corticotropin-releasing factor receptor 1	CRFR1	0.68	ND	ND	(*R*)-PZQ

Supplementary Table 1. Predictions of Docking to Pocket Classification/Activity ('dpc') Screen. The top docking candidates of two enantiomers of PZQ, (*R*)-PZQ and (*S*)-PZQ from the *in silico* screen against pocket docking models ('dpc') models, a subset of the full Molscreen panel. The drugs were docked and scored into ensemble 4D models of various targets assembled in the Pocketome database. Candidate target, accession code and docked enantiomer are listed together with predicted estimates of binding affinity ('Mol pKd') and hit probability prioritization ('pPvalue'). Strength of interaction is conveyed on a pseudocolor scale.
