# Supplementary material for: The anthelmintic praziquantel is a human serotoninergic G-protein-coupled receptor ligand
Source: Nat Commun. 2017 Dec 5;8:1910. doi: 10.1038/s41467-017-02084-0 (PMC5716991; doi:10.1038/s41467-017-02084-0)
Supplement: Supplementary file 2 — Description of Additional Supplementary Files [file 41467_2017_2084_MOESM2_ESM.pdf]

Description of Additional Supplementary Files

File Name: Supplementary Movie 1
Description: PZQ causes a rapid paralysis of adults schistosome worms. Video showing basal movement of schistosome worms in a petri dish (10x normal speed) before and after addition of PZQ (1µM).
